# Adaptive c-Met-PLXDC2 Signaling Axis Mediates Cancer Stem Cell Plasticity to Confer Radioresistance-associated Aggressiveness in Head and Neck Cancer

**DOI:** 10.1158/2767-9764.CRC-22-0289

**Published:** 2023-04-19

**Authors:** Liwei Lang, Fanghui Chen, Yamin Li, Chloe Shay, Fan Yang, Hancai Dan, Zhuo G. Chen, Nabil F. Saba, Yong Teng

**Affiliations:** 1 Department of Oral Biology and Diagnostic Sciences, Georgia Cancer Center, Augusta University, Augusta, Georgia.; 2 Department of Hematology and Medical Oncology, Winship Cancer Institute, Emory University, School of Medicine, Atlanta, Georgia.; 3 Department of Pharmacology, SUNY Upstate Medical University, Syracuse, New York.; 4 Wallace H. Coulter Department of Biomedical Engineering, Georgia Institute of Technology and Emory, University, Atlanta, Georgia.; 5 Department of Pathology, University of Maryland Greenebaum Comprehensive Cancer Center, University of Maryland School of Medicine, Baltimore, Maryland.

## Abstract

Radiotherapy plays an essential role in the treatment of head and neck squamous cell carcinoma (HNSCC), yet radioresistance remains a major barrier to therapeutic efficacy. A better understanding of the predominant pathways determining radiotherapy response could help develop mechanism-informed therapies to improve cancer management. Here we report that radioresistant HNSCC cells exhibit increased tumor aggressiveness. Using unbiased proteome profiler antibody arrays, we identify that upregulation of c-Met phosphorylation is one of the critical mechanisms for radioresistance in HNSCC cells. We further uncover that radioresistance-associated HNSCC aggressiveness is effectively exacerbated by c-Met but is suppressed by its genetic knockdown and pharmacologic inactivation. Mechanistically, the resulting upregulation of c-Met promotes elevated expression of plexin domain containing 2 (PLXDC2) through activating ERK1/2-ELK1 signaling, which in turn modulates cancer cell plasticity by epithelial–mesenchymal transition (EMT) induction and enrichment of the cancer stem cell (CSC) subpopulation, leading to resistance of HNSCC cells to radiotherapy. Depletion of PLXDC2 overcomes c-Met–mediated radioresistance through reversing the EMT progress and blunting the self-renewal capacity of CSCs. Therapeutically, the addition of SU11274, a selective and potent c-Met inhibitor, to radiation induces tumor shrinkage and limits tumor metastasis to lymph nodes in an orthotopic mouse model. Collectively, these significant findings not only demonstrate a novel mechanism underpinning radioresistance-associated aggressiveness but also provide a possible therapeutic strategy to target radioresistance in patients with HNSCC.

**Significance:**

This work provides novel insights into c-Met-PLXDC2 signaling in radioresistance-associated aggressiveness and suggests a new mechanism-informed therapeutic strategy to overcome failure of radiotherapy in patients with HNSCC.

## Introduction

Head and neck squamous cell carcinoma (HNSCC) is the sixth most common cancer worldwide with a poor prognosis and minimal improvement in survival in the advanced stages (1, 2). Fractionated radiotherapy with ionizing radiation (IR) remains a mainstay of treatment for a great majority of patients with HNSCC. A standard dose of 70 Gy in 35 fractions is usually given over 7 weeks. Although radiotherapy eradicates a large fraction of tumor cells, selected groups of tumor cells may acquire radioresistance resulting in poor patient prognosis and survival (3). The median overall survival (OS) for patients with HNSCC with relapse after radiotherapy is only up to 9 months, and 10%–25% of patients are diagnosed with metastasis after radiotherapy (1, 4, 5). It is therefore one of the foremost challenges to develop effective therapeutic options to increase the efficacy of radiotherapy and even circumvent radioresistance during treatment for HNSCC. Therefore, the key molecular players and underlying molecular mechanisms responsible for radioresistance must be thoroughly investigated.

Pathways that have been implicated in the acquisition of radioresistance in HNSCC include EGFR, p53, and PI3K/Akt/mTOR signaling (6, 7). Alterations in these molecules and their mediated intracellular pathways are largely related to cell proliferation and apoptosis, hypoxic conditions inside the tumor microenvironment (TME), and DNA damage and repair. Apart from these pathways, epithelial–mesenchymal transition (EMT), a dynamic two-way process in the initiation and progression of cancer, is also associated with radioresistance (8, 9). Previous studies demonstrated that radioresistant cancer cells surviving from radiation commonly display an EMT phenotype with an upregulation of mesenchymal markers (e.g.*,* vimentin and N-cadherin) and a downregulation of epithelial markers (e.g., E-cadherin), leading to augmented capacity for cancer cell self-renewal and invasive properties and eventually promoting tumor aggressiveness (10, 11).

Accumulating evidence has established a key role of nonmutational resistance mechanisms underlying radiotherapy. Such nonmutational processes are largely driven by tumor cell plasticity (8, 12, 13). At least one source of tumor cell plasticity is EMT and its associated emergence of dedifferentiated cells with cancer stem cell (CSC)-like properties. In the current study, we identify a novel c-Met–mediated mechanism that is mediated by radiotherapy in HNSCC. We report for the first time that c-Met confers radioresistance in HNSCC via plexin domain containing 2 (PLXDC2), a transmembrane receptor for pigment epithelium-derived factor, which in turn triggers cancer cell plasticity by promoting EMT and enrichment of dedifferentiated cells with CSC-like properties. These novel findings suggest that dysregulation of c-Met-PLXDC2 signaling is likely a major contributing factor in the acquisition of radioresistance in HNSCC.

## Materials and Methods

### Cell Lines and Radioresistant Cells

HN6 cells were a gift from Dr. W. Andrew Yeudall in 2016 and maintained in our lab (14, 15). CAL27 cells were obtained from ATCC in 2020. All cells were used for experiments before passage 10 and cultured in DMEM/F-12 medium containing 10% FCS at 37°C in a humidified incubator supplied with 5% CO_2_. Luciferase stable HN6 and CAL27 cells were generated by transduction of pGL4.5 vector (Promega) and selection with hygromycin for 6 weeks. HN6 and CAL27 radioresistant cells were generated as we described previously (7). Briefly, luciferase-containing HN6 and CAL27 cells were irradiated at a dose of 2 Gy. The irradiated cells were cultured, split 1:3, and exposed to another cycle of 2 Gy at 80% confluence. This process was repeated for a cumulative total of 40 and 60 Gy of IR, and the resulting cell population was expanded and maintained in the same manner as the parental cells. The radioresistant cells were confirmed for cell viability by comparison with their parental cells at the different doses of IR. All cell lines were not genetically authenticated but were routinely screened for *Mycoplasma* contamination by MycoAlert *Mycoplasma* Detection Kit (Lonza).

### Reagents, Antibodies, and Standard Assays

Antibodies that recognize c-Met, p-c-Met (Tyr1234/1235), ERK1/2, p-ERK1/2, AKT, p-AKT, E-cadherin, and vimentin were purchased from Cell Signaling Technology. Antibodies against PLXDC2 and ELK1 were ordered from Proteintech and Abcam, respectively. The c-Met inhibitor SU11274 and β-Actin antibody were obtained from Sigma-Aldrich. The secondary antibodies were purchased from Invitrogen. PE mouse anti-human CD133 and FITC mouse anti-human CD44 were obtained from BD Biosciences and used for flow cytometry. The c-Met inhibitor foretinib and crizotinib, AKT inhibitor AZD5363, and ERK1/2 inhibitor SCH772984 were obtained from SelleckChem. Cell viability was measured by alamarBlue Cell Viability Reagent (Thermo Fisher Scientific) following the indicated treatments. Western blotting, wound healing assays, cell proliferation, and colony formation assays were carried out as described previously (15, 16).

### Patients, Tissue Specimens, and IHC

The selected patients had been diagnosed pathologically with HNSCC and had local recurrence after the completion of radiotherapy. Adequate histologic specimens containing tumor cells were collected in paired tissue samples from both preradiotherapy and local recurrence sites. These paraffin-embedded paired tumor specimens were obtained from the Head and Neck Satellite Tissue Bank of Emory University (Atlanta, GA). The tissue collection was approved by the Emory Institutional Review Board and conducted in compliance with ethical standards and good clinical practice. Informed consents were obtained from patients. IHC was performed as described previously (17, 18). In brief, IHC for PLXDC2 was performed by incubating the sections with the primary antibody against PLXDC2, and images were captured by a CCD camera (Olympus). The final immunoreactivity score was examined by three investigators who were blind to pathologic information by using the German semiquantitative scoring method as we described previously (18). Each specimen was scored for intensity (no staining  =  0; weak staining  =  1; moderate staining  =  2; strong staining  =  3) and for extent of stained cells (0%  =  0; 1%–24%  =  1; 25%–49%  =  2; 50%–74%  =  3; 75%–100%  =  4). Signal index (SI) was the product of the intensity score multiplied by the extent score. Consecutive sections were stained with hematoxylin and eosin (H&E) to help localize cancer tissues and adjacent normal epithelium.

### Phospho-receptor Tyrosine Kinase Profiling

The Proteome Profiler Human Phospho-receptor tyrosine kinase (RTK) Array Kit (R&D Systems) was used to determine the phospho-RTK profiling according to the manufacturer's protocol. Briefly, a total of 500 μg fresh protein was diluted and incubated overnight with nitrocellulose membranes dotted with duplicate spots for 49 anti-RTK and control antibodies. Bound phospho-RTKs were detected with a pan anti-phosphotyrosine antibody conjugated to horseradish peroxidase, and the data were digitized and subjected to ImageJ Fiji (version 1.2). By subtracting the background staining and normalizing to the positive controls on the same membrane, we obtained relative protein levels.

### *In Vitro* Tumorsphere Formation and Cell Invasion

Dissociated single HNSCC cells were plated on 6-well ultra-low attachment plates (Corning) at a density of 1 × 10^5^ cells per mL and grown in serum-free DMEM/F-12 medium with B27 (Invitrogen), EGF (20 ng/mL), basic FGF (20 ng/mL), and insulin (5 μg/mL; Peprotech). After a 2-week incubation, the number of spheres was counted using a Zeiss microscope. The average size of the randomly selected mammospheres (*n*  =  30) was calculated. For Matrigel invasion assays, cells were plated onto an 8‐mm invasion chamber covered with Matrigel (BD Biosciences) at a density of 5 × 10^4^ cells/well and incubated for 24 hours. The chemotactic invasion of cells was induced by 10% FBS placed in the lower chamber. The invasive cells were fixed with 1% glutaraldehyde and then stained with 0.5% crystal violet staining solution. Quantification was calculated after 1 hour solubilization in Triton X-100 solution and the relative absorbance was determined at 570 nm.

### RNA Sequencing and Data Analysis

Total RNA was isolated using TRIzol (Invitrogen) from radioresistant and parental CAL27 cells. RNA quality was analyzed using a 2100 Bioanalyzer (Agilent Technologies). Illumina sequencing was carried out by Novogene as described previously (19) for each sample. The bowtie parameter mismatch was 2. Clean data were mapped back onto the assembled transcriptome, and a read count for each gene was obtained from the mapping results. Differentially expressed genes (≥ ±2-fold, *P* < 0.05) were subjected to Gene Ontology enrichment and Kyoto Encyclopedia of Genes and Genomes pathway analysis. The detailed RNA sequencing (RNA-seq) information of this assay is available in GSE182763 deposited in the NIH Gene Expression Omnibus database.

### Gene Modifications

The pLKO.1-puro TRC short hairpin RNAs (shRNA) targeting GFP, c-Met, ELK1, or PLXDC2 were obtained from Horizon Discovery. shRNA plasmids, together with packaging/envelope plasmids psPAX2 and pMD2.G, were cotransfected into Lenti-Pac 293Ta cells (GeneCopoeia) using Lipofectamine 3000 (Invitrogen) following the manufacturer's instructions. Forty-eight hours after transfection, virus particles were collected and transfected into HN6 and CAL27 cells to generate stable knockdown cell lines. Cells transfected with shGFP were used as a negative control. Human c-Met expression plasmid was purchased from Sino Biological. The efficacy of knockdown or overexpression was evaluated by Western blotting.

### Three-dimensional Cell Cultures and qRT-PCR Analysis

For three-dimensional (3D) cell cultures, 2 × 10^5^ HN6 or CAL27 radioresistant cells were seeded in 48-well SeedEZ scaffold (Lena Bioscience) with complete medium. After a 5-day culture, cells growing in SeedEZ were exposed to 10 μmol/L SU11274 and 4 Gy IR alone or in combination. After an 8-day treatment, cell viability in SeedEZ scaffold was measured by alamarBlue at 545/590 nm ex/em, followed by phalloidin (Invitrogen) staining and imaging. For qRT-PCR analysis, reverse transcription of RNA to cDNA was conducted using iScript Advanced cDNA Synthesis Kit (Bio-Rad), and qRT-PCR was performed with FastStart Universal SYBR Green Master (Roche) on the StepOne Plus Real Time PCR System (Applied Biosystems). The relative expression of target genes was determined using the comparative CT (cycle threshold) method and normalized with housekeeping gene β-actin. Primer information is listed in Supplementary Table S1.

### Chromatin Immunoprecipitation q-PCR

Chromatin immunoprecipitation (ChIP) assays were performed using a ChIP assay kit (MilliporeSigma) as we described previously (20, 21). In brief, chromatin from cells was cross-linked with 1% formaldehyde for 10 minutes at room temperature, sheared to an average size of approximately 500 bp and then immunoprecipitated with a control IgG or anti-ELK1 antibody. Each immunoprecipitated DNA sample was quantified by qPCR using the primers (F: 5′- TTAAACTCTCGCTTTCGCCCC-3′; R: 5′-CCAGAGCCGGTTCGGTTAC-3′) that were designed to amplify a proximal promoter region containing putative ELK1 binding sites on the *PLXDC2* promoter. All samples were run in triplicate, and results were averaged after normalization to the input.

### Animal Study

Six-week-old NOD.Cg-*Prkdcscid Il2rgtm1Wjl/SzJ* (NSG) mice were purchased from the Jackson Laboratory. All animal experiments were approved by the Institutional Animal Care and Use Committee of Augusta University (Augusta, GA). To generate an orthotopic xenograft tongue tumor model, 5 × 10^4^ luciferase-containing CAL27 cells were suspended in 50 μL of PBS/Matrigel (3:1) and injected into the anterior portion of the tongue of NSG mice. On day 10 after injection, mice were randomized to receive different treatments: vehicle (PBS), SU11274, IR, or SU11274 plus IR. For IR, when tumor-bearing mice were anesthetized with isoflurane, the tongue with tumor was located by CT scan and a total dose of 12 Gy IR was delivered using 4 Gy fractions per day for 3 days in the Small Animal Radiation Research Platform (7). For drug experiments, SU11274 at the dose of 10 mg/kg body weight was administered by intravenous injection every other day for 2 weeks. Mice were then intraperitoneally injected with d-luciferin bioluminescent substrate (Sigma), and tumor growth and metastasis were measured by bioluminescent luciferase signal using a Xenogen IVIS-200 In Vivo Imaging System (PerkinElmer). Afterward, mice were sacrificed and the xenografts and major organs (including the heart, intestine, kidney, liver, lung, and spleen) were excised for histopathologic analysis with H&E staining and IHC with E-cadherin and Ki67 antibodies. The final immunoreactive score was examined by three investigators who were blind to pathological information. At least 10 random microscopic fields were captured per sample, and signal intensity was semiquantified using ImageJ Fiji (version 1.2).

### Bioinformatics and Statistical Analysis

RNA-seq data of The Cancer Genome Atlas (TCGA) head and neck cancer cohort were downloaded from UCSC Xena website (https://xenabrowser.net/), and FPKM (fragments per kilobase per million) format data were transformed into TPM format (transcripts per kilobase million). Statistical software GraphPad Prism 9 was used for all statistical analyses. All data were presented as mean ± SDs. Comparisons among multiple groups were performed using the one-way ANOVA test. Differences were considered statistically significant when *P* < 0.05.

### Data Availability Statement

All data generated or analyzed in this study are available within the article and its Supplementary Data.

## Results

### Radioresistant HNSCC Cells Exhibit Increased Tumor Aggressiveness

**FIGURE 1:**
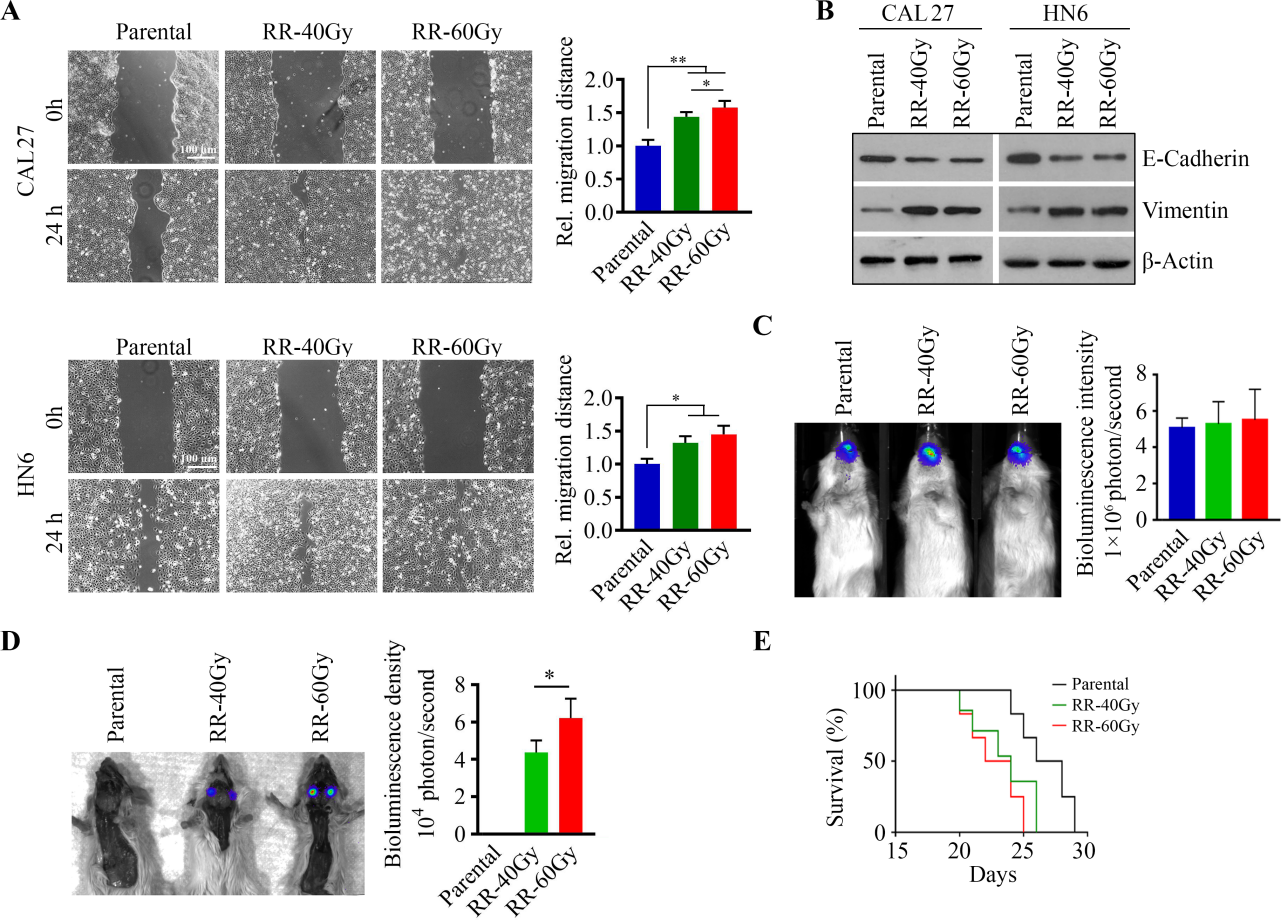
Radioresistant HNSCC cells exhibit enhanced migration *in vitro* and metastasis *in vivo*. **A,** The migration potential of radioresistant and parental CAL27 and HN6 cells determined by wound healing assays. Representative phase-contrast images and quantitative data from three independent experiments are shown in the left and right, respectively. **B,** Increased vimentin and decreased E-cadherin in radioresistant CAL27 and HN6 cells compared with their parental cells. **C,** Bioluminescence images of tongue tumors in NSG mice implanted with radioresistant or parental CAL27 cells 3 weeks after cell inoculation. Quantitative data of bioluminescence intensity of tongue tumors are shown in the right (*n* = 5/group). **D,** Bioluminescence images of tumor metastasis in lymph nodes in NSG mice implanted with radioresistant or parental CAL27 cells 3 weeks after cell inoculation. Quantitative data of bioluminescence density in lymph nodes are shown in the right (*n* = 5/group). **E,** Survival curve of mice receiving radioresistant or parental CAL27 cells (*n* = 5/group). *, *P* < 0.05; **, *P* < 0.01.

Our previous study has shown that CAL27 and HN6 cells are IR-sensitive HNSCC cells (7). To understand the potential molecular mechanisms underpinning acquired HNSCC radioresistance, we generated radioresistant cells by exposing CAL27 and HN6 cells to a cumulative 40 Gy (RR-40Gy) and 60 Gy (RR-60Gy) of fractionated IR over 6 weeks, a protocol commonly used clinically. Cell viability assays confirmed that radioresistant cells were more tolerant to IR compared with their corresponding parental cells (Supplementary Fig. S1). Radioresistance was stable and was retained after more than 20 passages. There was no noticeable difference in cell proliferation between radioresistant and parental CAL27 and HN6 cells 3 days after incubation (Supplementary Fig. S2). However, relatively higher levels of migration potential were seen in both radioresistant cell lines as revealed by increased cell motility in wound healing assays (Fig. 1A). Consistently, the levels of mesenchymal marker vimentin were increased in these radioresistant cells, accompanied by a reduction in the levels of epithelial marker E-cadherin, as compared with the parental cells (Fig. 1B).

To further investigate whether radioresistance confers HNSCC metastasis, radioresistant and parental CAL27 cells were individually injected into the anterior tongue of NSG mice. Three weeks after inoculation, there was no change in tumor growth between the two groups of mice receiving radioresistant or parental cells (Fig. 1C). Intriguingly, radioresistant CAL27 cells, but not parental cells, developed cervical lymph node metastases (LNMet) in mice after 3 weeks as shown by the bioluminescence signal in lymph nodes (Fig. 1D). In line with the results from *in vitro* wound healing assays (Fig. 1A), CAL27:RR-60Gy cells became more aggressive compared with CAL27:RR-40Gy cells as more CAL27:RR-60Gy cells were found to metastasize to mouse lymph nodes (Fig. 1D). OS was quite similar between these two groups (implanted with CAL27:RR-40Gy or CAL27:RR-60Gy cells) but significantly shorter than in the control group implanted with parental cells (Fig. 1E). These findings strongly suggest the association of metastatic potential with the acquisition of radioresistance in HNSCC.

### Increased c-Met Phosphor-activation is Associated with Radioresistance in HNSCC Cells

**FIGURE 2:**
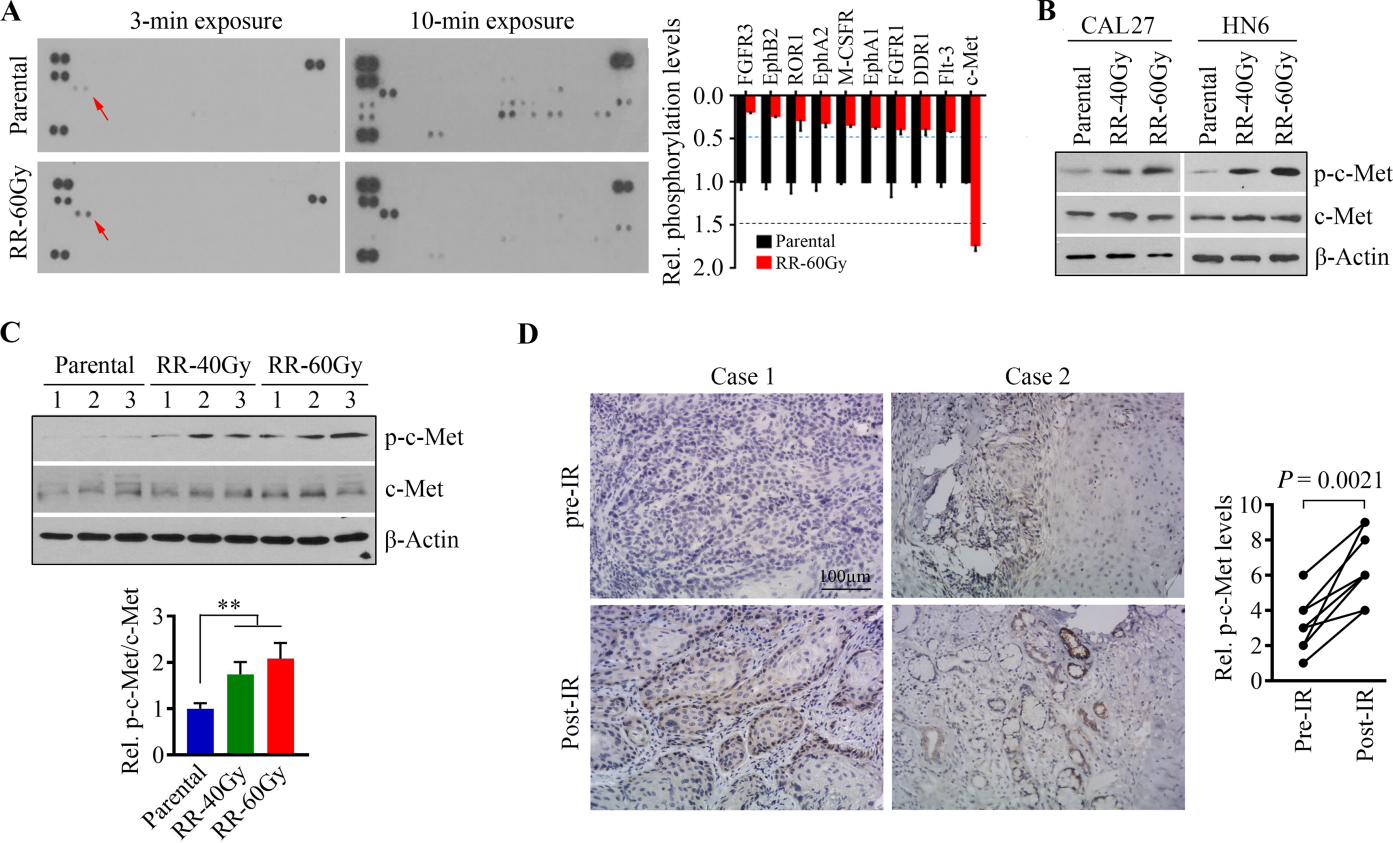
The phosphorylation levels of c-Met are enriched when HNSCC cells develop acquired radioresistance. **A,** Results from Human Phospho-RTK array showing c-Met as the most activated RTK in radioresistant CAL27 cells. Representative images (with the short and long exposure time) and quantitative data from two independent experiments are shown in the left and right, respectively. **B,** Increased phospho-c-Met levels in radioresistant CAL27 and HN6 cells confirmed by Western blotting. **C,** Protein and phosphorylation levels of c-Met in xenograft tongue tumors derived from radioresistant or parental CAL27 cells. Representative western blotting images and quantitative data (*n* = 10 mice/group) are shown in the left and right, respectively. **D,** Immunostaining of p-c-Met in paired primary HNSCC patient tumor tissues (*n* = 8) obtained before and after IR. Patients were considered nonresponders to radiotherapy when IR is unable to reduce its volume or when a recurrence occurs after a possible regression. SI  =  positive staining ×  intensity score. *, *P* < 0.05; **, *P* < 0.01.

Alterations in the expression and/or activation of membrane-localized oncogenic RTKs have been associated with cancer radioresistance (22, 23). We applied the Proteome Profiler Human Phospho-RTK Array Kit to identify the key RTK-mediating radioresistance in HNSCC cells. The phosphorylation of nine RTKs (of total 49 RTKs) was dramatically decreased in radioresistant CAL27 cells compared with their parental cells (Fig. 2A). However, much higher levels of phospho-c-Met were detected in radioresistant CAL27 cells (Fig. 2A). Western blotting analysis confirmed the increase of phosphor-activation of c-Met in radioresistant CAL27 and HN6 cells, with a slight increase in c-Met protein (Fig. 2B). The same tendency in c-Met expression and phosphorylation was found in the xenograft tumors derived from radioresistant and parental CAL27 cells (Fig. 2C). To understand the clinical relevance, we collected eight paired head and neck tumor tissues from both preradiotherapy and local recurrence sites and performed IHC with phospho-c-Met. This analysis showed significantly increased c-Met phosphorylation level in recurrent tumor tissues after IR (Fig. 2D). These observations support the notion that modulation of c-Met signaling is associated with the sensitivity of HNSCC cells to radiation.

### Ablation of c-Met Attenuates the Increased Aggressiveness in Radioresistant HNSCC Cells

**FIGURE 3:**
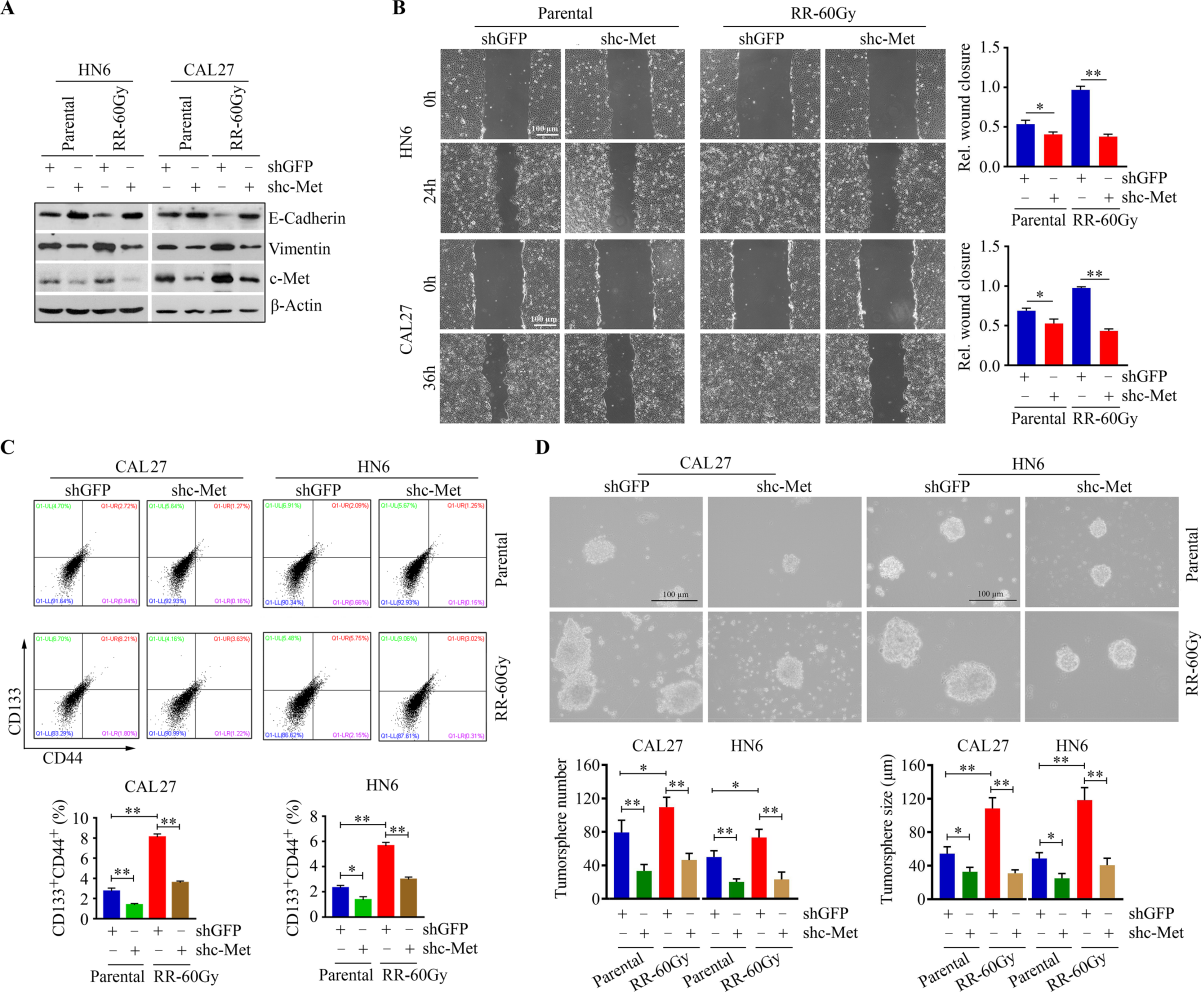
Loss of c-Met function attenuates radioresistance-associated aggressiveness in HNSCC cells. **A,** Effect of c-Met knockdown on the levels of E-cadherin and vimentin in radioresistant and parental CAL27 and HN6 cells. **B,** Effect of c-Met knockdown on migration in radioresistant and parental CAL27 and HN6 cells. Representative phase-contrast images and quantitative data from three independent experiments are shown in the left and right, respectively. **C,** Effect of c-Met knockdown on CSC population in radioresistant and parental CAL27 and HN6 cells. **D,** Effect of c-Met knockdown on tumorsphere formation in radioresistant and parental CAL27 and HN6 cells. In C and D, representative images and quantitative data from three independent experiments are shown in the top and bottom, respectively. shGFP: a control shRNA against GFP; shc-Met: a specific shRNA against the c-Met gene; *, *P* < 0.05; **, *P* < 0.01.

To explore how c-Met contributes to radioresistance-associated tumor aggressiveness, we depleted c-Met using an shRNA strategy in radioresistant and parental CAL27 cells. Knockdown of c-Met upregulated E-cadherin and downregulated vimentin in the parental cells (Fig. 3A), leading to a dramatic decrease in cell migration (Fig. 3B). Loss of c-Met expression significantly attenuated the increased vimentin and cell motility and restored the expression levels of E-cadherin (Fig. 3A and B). This tendency was also found in radioresistant and parental HN6 cells with or without c-Met knockdown (Fig. 3A and B), suggesting that c-Met–mediated EMT contributes to HNSCC cell migration associated with radioresistance.

CSCs are a distinct subpopulation within a tumor, which are well recognized as one of the radioresistant cell types in radiotherapy (4). To test whether c-Met supports the repopulation of CSCs in radioresistant HNSCC cells, we determined the number of CSCs in radioresistant and parental CAL27 and HN6 cells with or without c-Met depletion. Flow cytometry analysis using CSC biomarkers indicated more CSCs in the radioresistant cells compared with the parental cells (Fig. 3C), which was confirmed by the evidence of increased number and size of tumorspheres (Fig. 3D). Nevertheless, knockdown of c-Met robustly suppressed the CSC population and impaired the self-renewal capacity in both radioresistant and parental cells (Fig. 3C and D). These findings suggest that radioresistance-associated HNSCC aggressiveness is effectively exacerbated by c-Met but could be suppressed by its inactivation.

### Inactivation of c-Met by SU11274 Abrogates the Increased Aggressiveness in Radioresistant HNSCC Cells

**FIGURE 4:**
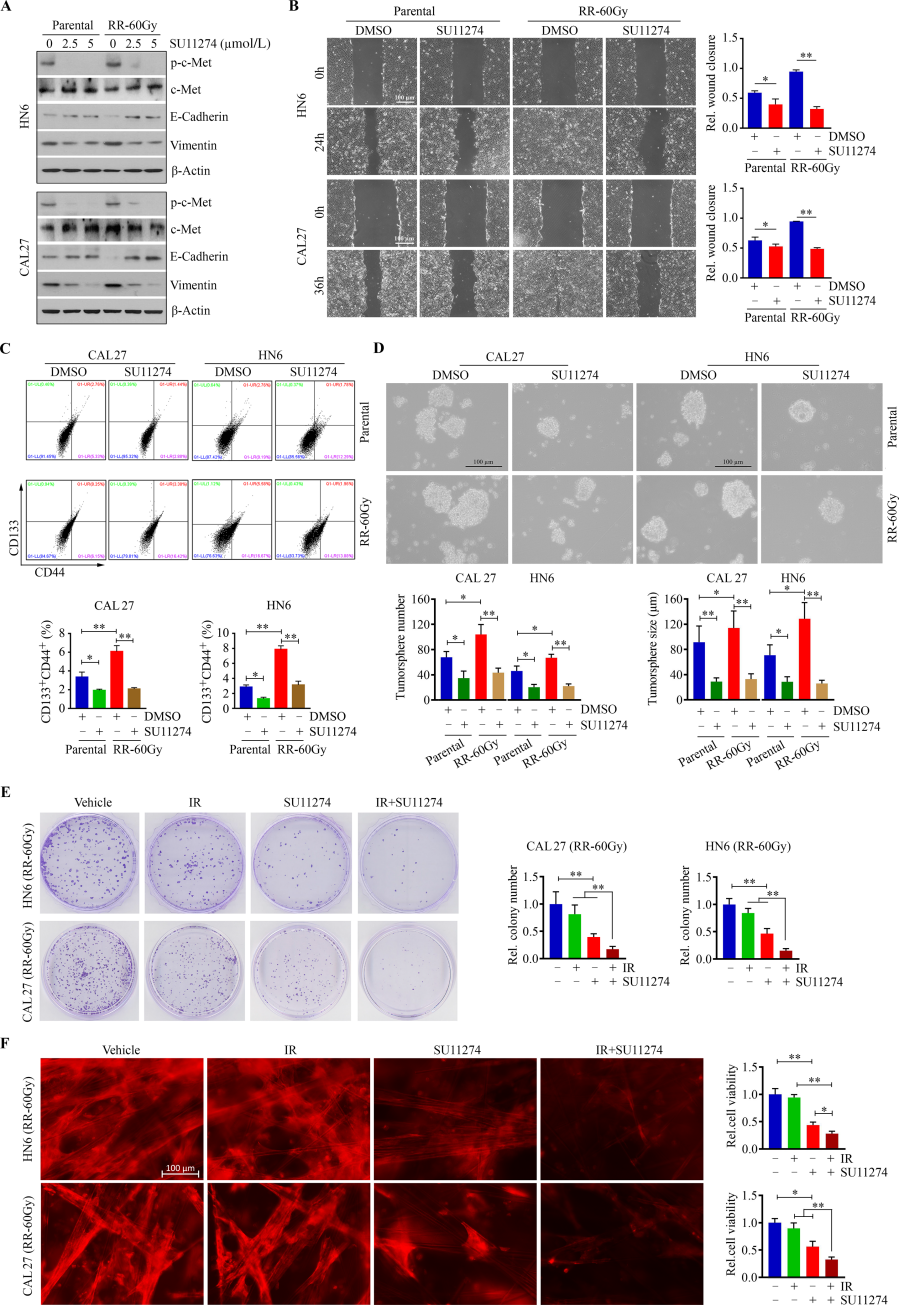
SU11274 abrogates radioresistance-associated aggressiveness in HNSCC cells. **A,** Effect of SU11274 (at two different doses of 2.5 and 5 μmol/L) on the expression of E-cadherin and vimentin in radioresistant and parental CAL27 and HN6 cells. **B,** Effect of 2.5 μmol/L SU11274 on migration in radioresistant and parental CAL27 and HN6 cells. Representative phase-contrast images and quantitative data from three independent experiments are shown in the left and right, respectively. **C,** Effect of 2.5 μmol/L SU11274 on the CSC population in radioresistant and parental CAL27 and HN6 cells on day 3 after treatment. **D,** Effect of 2.5 μmol/L SU11274 on tumorsphere formation in radioresistant and parental CAL27 and HN6 cells after a 2-week treatment. In **C** and **D,** representative images and quantitative data from three independent experiments are shown in the top and bottom, respectively. **E,** Effect of 2.5 μmol/L SU11274 on colony formation of radioresistant CAL27 and HN6 cells determined on day 12 after treatment. Representative images and quantitative data are shown in the left and right, respectively. **F,** Effect of 2.5 μmol/L SU11274 on viability of radioresistant CAL27 and HN6 cells in 3D SeedEZ scaffold determined on day 8 after treatment. Representative images of phalloidin staining and quantitative data of cell viability measured by alamarBlue are shown in the left and right, respectively. In B–F, quantitative data were obtained from three independent experiments. *, *P* < 0.05; **, *P* < 0.01.

SU11274 is an ATP-competitive inhibitor of c-Met showing superior selectivity toward c-Met versus other RTKs (e.g., PGDFRβ, EGFR, or FGFR1) compared with other c-Met inhibitors (24, 25). We screened three c-MET inhibitors (SU11274, foretinib, and crizotinib) in CAL27 cells at 2.5 μmol/L for 24 hours and compared their inhibitory effects on c-Met activation. This assay showed that SU11274 is the most effective inhibitor blocking c-Met phosphorylation in both cell lines examined (Supplementary Fig. S3). On the basis of this, we treated radioresistant and parental CAL27 and HN6 cells with or without 2.5 μmol/L SU11274. Consistent with c-Met genetic knockdown, SU11274 abrogated increased c-Met phosphorylation and cell migration in radioresistant CAL27 and HN6 cells (Fig. 4A and B). Moreover, SU11274 treatment led to a reversal of EMT biomarkers (Fig. 4A and B) and a suppression of the CSC population and tumorsphere formation in both radioresistant HNSCC cells (Fig. 4C and D). Moreover, colony formation assay further showed that the addition of SU11274 to IR abolished cell clonogenicity more dramatically than either of single treatment in both resistant HNSCC cell lines (Fig. 4E).

SeedEZ 3D scaffold is made by completely inert and transparent glass fibers, which can promote cell–cell interaction and cell network formation efficiently. To determine the combined effect of SU11274 and IR on cell growth and viability in 3D cell cultures, we cultured radioresistant CAL27 and HN6 cells in SeedEZ 3D scaffold, followed by treatment with 2.5 μmol/L SU11274, 4 Gy IR alone or in combination. SU11274, but not IR, reduced cell viability to almost half in 3D culture, while this inhibitory effect was more pronounced with the combination treatment of SU11274 and IR (Fig. 4F), suggesting that synergy of SU11274 and IR may greatly potentiate the radiosensitivity of HNSCC cells.

**FIGURE 5:**
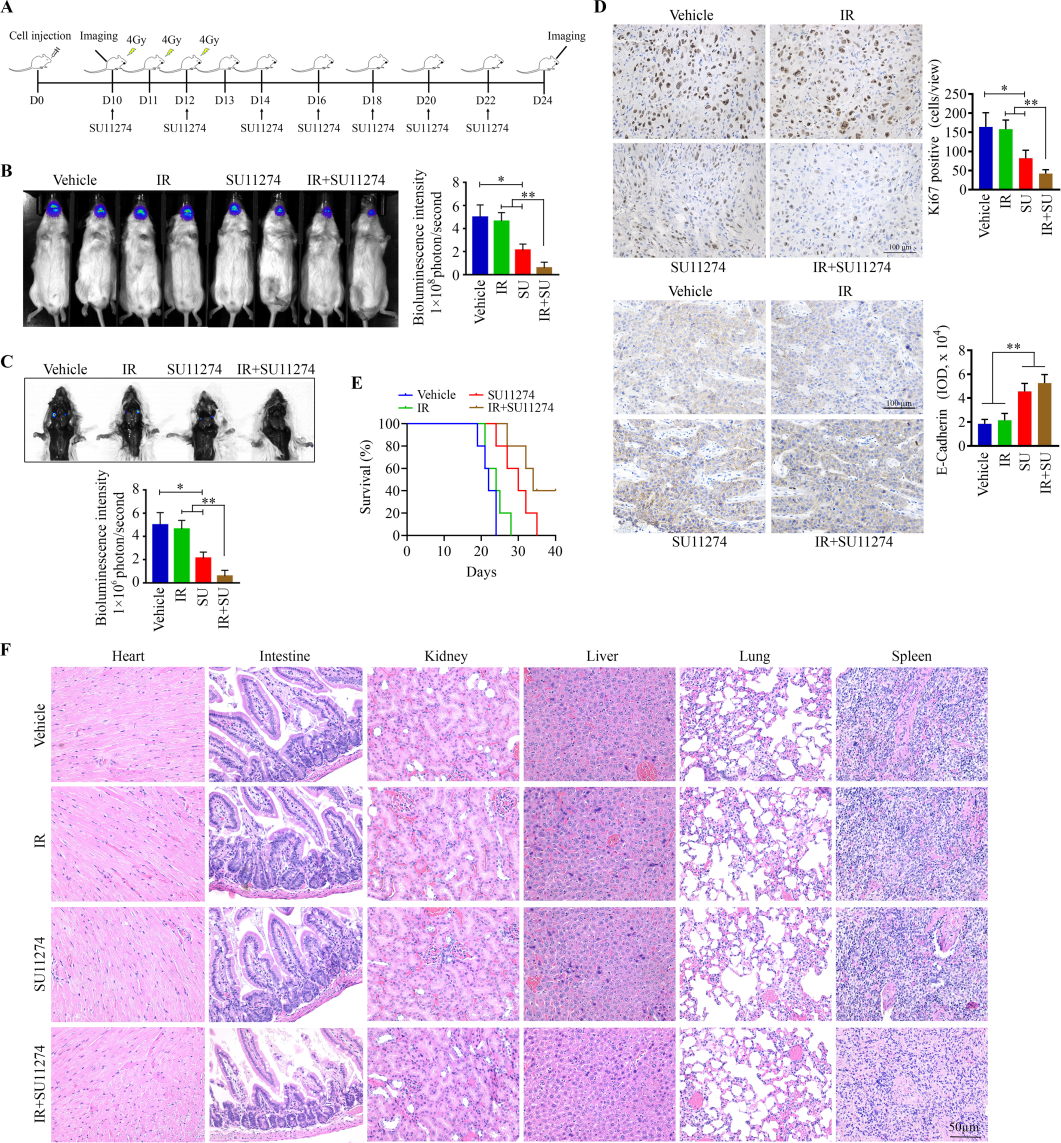
SU11274 potentiates treatment efficacy of IR in radioresistant tongue tumors in orthotopic xenograft mice. **A,** Timeline of experimental procedures *in vivo*. CAL27:RR-60Gy–bearing mice (*n* = 5/group) received IR and/or SU11274 treatment on day 10 (D10) after cell inoculation and were sacrificed on D24. **B,** Bioluminescence images of tongue tumors in mice receiving IR and/or SU11274 treatment on D24. **C,** Bioluminescence images of LNMets in mice receiving IR and/or SU11274 treatment on D24. **D,** Immunostaining of Ki67 and E-cadherin in tumor xenografts from mice receiving IR and/or SU11274 treatment. Representative IHC images and quantitative data (*n* = 5) are shown in the left and right, respectively. **E,** Mouse survival curves for groups receiving IR and/or SU11274 treatment (log-rank test). **F,** Histology of major organs from mice receiving IR and/or SU11274 treatment. *, *P* < 0.05; **, *P* < 0.01.

Next, we evaluated the therapeutic efficacy of SU11274 in combination with IR *in vivo*. NSG mice were orthotopically inoculated with CAL27:RR-60Gy to form tongue xenografts. When the tongue tumors were established (on day 10), mice were randomized into four groups to receive the following treatments: vehicle, local IR to the tongue, SU11274, or SU11274 combined with local IR (Fig. 5A). Fractionated IR treatment alone for total 12 Gy did not lead to a significant reduction in tongue tumor growth and LNMets within 12 days postexposure, as measured by bioluminescence (Fig. 5B and C). However, reduced tumor size and LNMets were seen in mice that received SU11274 treatment, which was more pronounced in response to the combined SU11274 and IR treatment (Fig. 5B and C). IHC analysis further indicated that this combination suppressed cell proliferation more efficiently than either treatment alone as revealed by the lowest number of Ki67-positive cells in xenograft tumor tissues among all treatment groups (Fig. 5D). At molecular level, SU11274 treatment alone or in combination with IR enhanced E-cadherin expression in radioresistant CAL27 cell-derived tumors (Fig. 5D). The survival outcomes of combined and single treatment were also recorded and evaluated, which indicated that the mice receiving the combination treatment survived longer than mice receiving single treatment (Fig. 5E). We also collected major organs (the heart, intestine, kidney, liver, lung, and spleen) from treated mice and examined the pathologic changes. H&E analysis showed there were no detectable morphologic changes in these major organs upon single or combined treatment (Fig. 5F), suggesting that SU11274 has no or little systemic toxicity. Targeting c-Met could be developed into a promising treatment approach to overcome radioresistance in HNSCC.

### c-Met Contributes to Radioresistence-associated Aggressiveness Through Regulating PLXDC2-mediated Tumor Cell Plasticity in HNSCC Cells

**FIGURE 6:**
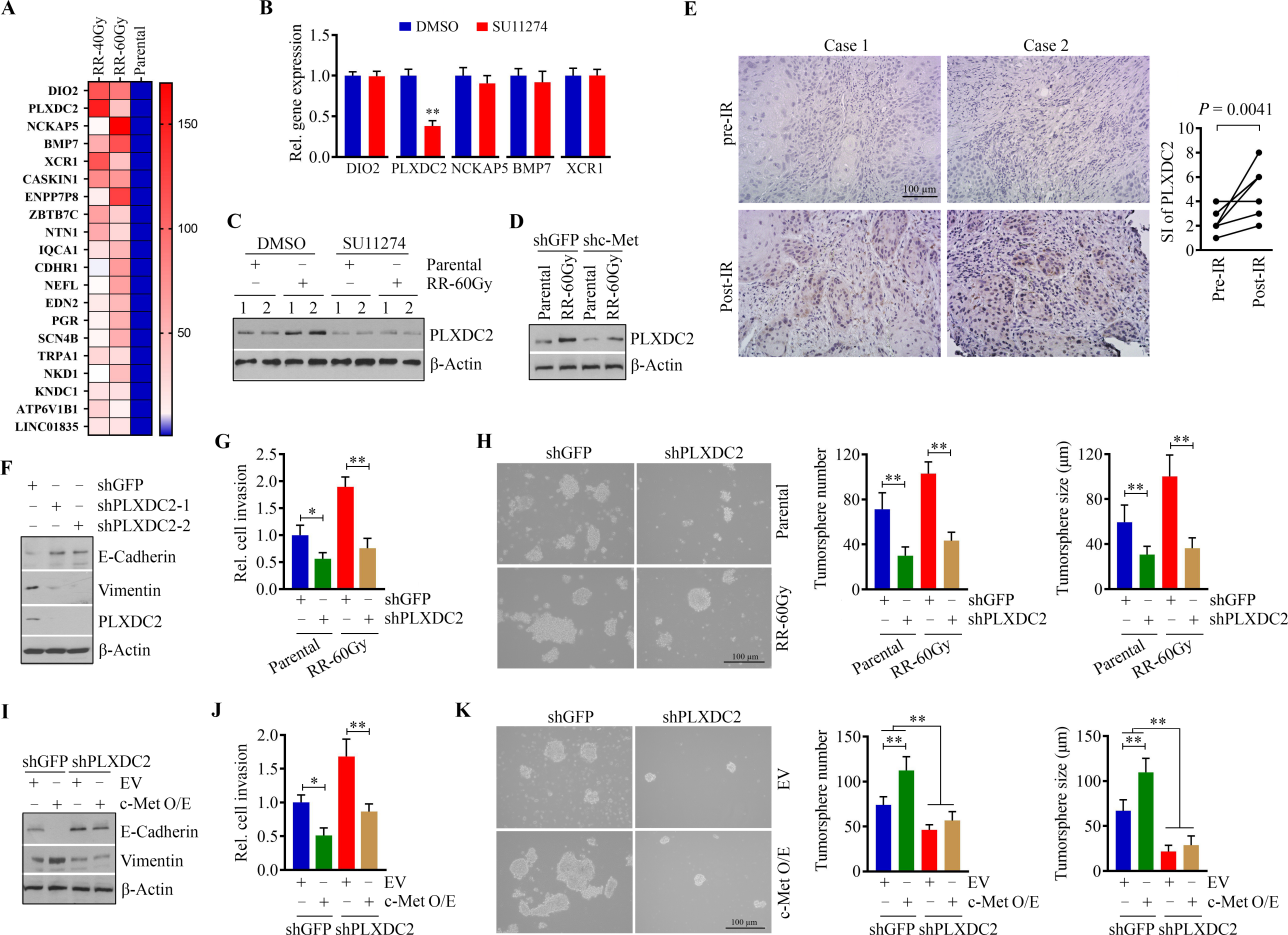
c-Met upregulates PLXDC2 in HNSCC cells to confer radioresistence-associated aggressiveness. **A,** Heatmap showing the top 20 upregulated genes in radioresistant CAL27 cells (RR-40Gy and RR-60Gy) compared with the parental cells based on RNA-seq data. **B,** Expression of the top five upregulated genes in CAL27 cells in the presence or absence of 2.5 μmol/L SU11274. **C,** Protein levels of PLXDC2 in radioresistant and parental CAL27 cells in the presence or absence of 2.5 μmol/L SU11274. **D,** Protein levels of PLXDC2 in radioresistant and parental CAL27 cells with or without c-Met knockdown. **E,** Immunostaining of PLXDC2 in paired primary HNSCC patient tumor tissues (*n* = 8) obtained before and after radiotherapy. Patients were considered non-responders to radiotherapy when IR is unable to reduce its volume or when a recurrence occurs after a possible regression. SI  =  positive staining ×  intensity score. **F,** Protein levels of E-cadherin and vimentin in PLXDC2 knockdown and control CAL27 cells. **G,** Effect of PLXDC2 knockdown on cell invasion in radioresistant and parental CAL27 cells. **H,** Effect of PLXDC2 knockdown on tumorsphere formation in radioresistant and parental CAL27 cells. **I,** Protein levels of E-cadherin and vimentin in c-Met overexpression and control CAL27 cells with or without PLXDC2 knockdown. **J,** Effect of PLXDC2 knockdown on cell invasion in c-Met overexpression and control CAL27 cells with or without PLXDC2 knockdown. **K,** Effect of PLXDC2 knockdown on c-Met–mediated tumorsphere formation in CAL27 cells. In G, H, J, and K, representative images and quantitative data from three independent experiments are shown in the left and right, respectively. shGFP: a control shRNA against GFP; shc-Met: a specific shRNA against the c-Met gene; shPLXDC2-1 and shPLXDC2-2: two different shRNAs against the PLXDC2 gene; EV: empty vector; c-Met O/E: c-Met overexpression vector. *, *P* < 0.05; **, *P* < 0.01.

To understand the mRNA regulatory network associated with the acquisition of radioresistance in an unbiased manner, we performed RNA-seq using RNA samples extracted from radioresistant and parental CAL27 cells. RNA-seq data revealed that DIO2, PLXDC2, NCKAP5, BMP7, and XCR1 are the top five upregulated genes in radioresistant CAL27 cells (vs. the parental cells; Fig. 6A), which were validated by qRT-PCR (Supplementary Fig. S4A). Elevated expression of these five genes was also observed in radioresistant HN6 cells compared with parental cells (Supplementary Fig. S4B). To explore whether c-Met activation affects the expression of these genes in HNSCC cells, we treated CAL27 cells with or without 2.5 μmol/L SU11274 before subjecting them to qRT-PCR. This analysis revealed *PLXDC2* as the only gene downregulated in CAL 27 cells in the presence of SU11274 (Fig. 6B). Intriguingly, increased PLXDC2 in radioresistant CAL27 cells was significantly attenuated by SU11274 (Fig. 6C). Consistently, genetic knockdown of c-Met reduced PLXDC2 levels in radioresistant and parental CAL27 cells (Fig. 6D), suggesting blocking c-Met signaling suppresses PLXDC2 expression in HNSCC cells. Most importantly, comparison of PLXDC2 protein expression between paired pre- and post-IR tumor tissues from patients with HNSCC who did not respond to radiotherapy further demonstrated that PLXDC2 expression can be induced during treatment (Fig. 6E). Together with the data shown in Fig. 2D, our findings suggest upregulated c-Met-PLXDC2 signaling axis in acquired resistance.

To understand the role of PLXDC2 in c-Met–mediated radioresistance, we silenced it in CAL27 and HN6 cells using specific shRNAs. Knockdown of PLXDC2 upregulated the epithelial marker E-cadherin and downregulated the mesenchymal marker vimentin (Fig. 6F; Supplementary Fig. S5A), leading to a remarkable reduction in cell invasion (Fig. 6G; Supplementary Fig. S5B). Colony formation assays further showed that depletion of PLXDC2 reversed radioresistance in both CAL27 and HN6, as seen less colonies in PLXDC2 knockdown cells exposed to 4 Gy IR (Supplementary Fig. S6). Moreover, loss of PLXDC2 suppressed the self-renewal capacity of cancer cells as evidenced by a reduced number of tumorspheres in either radioresistant or parental CAL27 cells (Fig. 6H). Importantly, depleting PLXDC2 in c-Met overexpression CAL27 cells attenuated c-Met–mediated E-cadherin upregulation and vimentin downregulation (Fig. 6I), which was confirmed in HN6 cells (Supplementary Fig. S5C). In accordance with these observations, PLXDC2 knockdown also abrogated increased cell invasion and self-renewal ability in c-Met overexpression CAL27 cells (Fig. 6J and K). Collectively, these results demonstrate that c-Met-PLXDC2 plays a critical role in developing radioresistance in HNSCC.

### Upregulation of PLXDC2 in Radioresistant HNSCC Cells is Mediated via c-Met-ERK1/2-ELK Signaling

**FIGURE 7:**
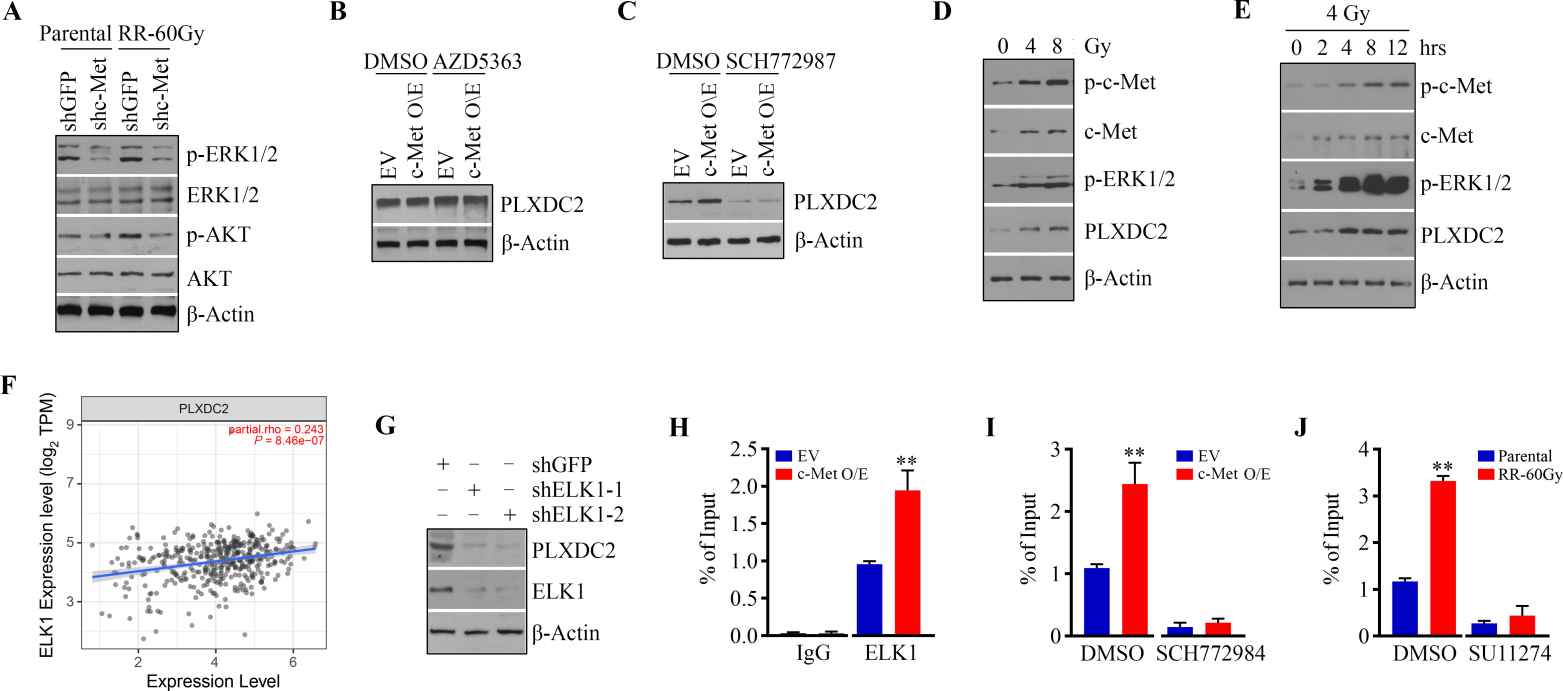
Elevated PLXDC2 expression in radioresistant HNSCC cells is mediated by c-Met–activated ERK1/2-ELK signaling. **A,** Effect of c-Met knockdown on inactivating ERK1/2 and AKT signaling in radioresistant and parental CAL27 cells. Effect of AKT inhibitor AZD5363 (**B**) or ERK1/2 inhibitor SCH772987 (**C**) on PLXDC2 expression in c-Met overexpression (c-Met O/E) and control (EV) CAL27 cells. **D,** The dose effect of IR on c-Met-ERK1/2-PLXDC2 signaling in CAL27 cells. **E,** Effect of IR at 4 Gy on c-Met-ERK1/2-PLXDC2 signaling in CAL27 cells within 12 hours after IR. **F,** The correlation between the expression of *ELK1* and *PLXDC2* genes illustrated in scatter plot from TCGA HPV(−) HNSCC cohort (*n* = 422). **G,** Effect of ELK1 knockdown on PLXDC2 expression in CAL27 cells. **H,** The binding of ELK1 protein on the *PLXDC2* gene promoter in c-Met overexpression and control CAL27 cells determined by ChIP-qPCR assays. **I,** Effect of SCH772984 on ELK1 binding on the *PLXDC2* gene promoter in c-Met overexpressing and control CAL27 cells determined by ChIP-qPCR assays. **J,** Effect of SU11274 on ELK1 binding on the *PLXDC2* gene promoter in radioresistant and parental CAL27 cells determined by ChIP-qPCR assays. In H, I, and J, quantitative data are obtained from three independent experiments. *, *P* < 0.05; **, *P* < 0.01.

We and others have demonstrated the enrichment of AKT and ERK1/2 signaling in radioresistant HNSCC cells (7, 26). Consistently, we observed upregulated AKT and ERK1/2 phosphorylation in radioresistant CAL27 cells compared with parental cells (Fig. 7A). Knockdown of c-Met inactivated both AKT and ERK1/2 signaling in parental CAL27 cells, and even attenuated these two pathways in radioresistant CAL27 cells (Fig. 7A). To determine whether AKT and/or ERK1/2 activation contributed to c-Met-PLXDC2 signaling regulation, we treated c-Met overexpression and control CAL27 cells with AKT inhibitor AZD5363 and ERK1/2 inhibitor SCH772987, respectively. Western blot assays showed that SCH772987, but not AZD5363, remarkably suppressed PLXDC2 expression in CAL27 cells regardless c-Met overexpression or not (Fig. 7B and C), suggesting the involvement of ERK1/2 signaling in c-Met–mediated PLXDC2 regulation. As shown in Fig. 7D, ERK1/2-PLXDC2 signaling axis was dose-dependently activated by IR, which was associated with c-MET phosphor-activation rather than its total expression levels. Moreover, dramatically increased c-Met-ERK1/2-PLXDC2 signaling was seen in CAL27 cells exposed to IR for 4 hours, which sustained at high level at least 12 hours after IR (Fig. 7E).

Next, we analyzed the *PLXDC2* gene promoter using PROMO, a virtual laboratory for the study of transcription factor binding sites in DNA sequences. ELK1 is an ETS-domain transcription factor playing a pivotal role in transducing extracellular signals into a transcriptional response through acting as a downstream target of ERK1/2 signaling (27, 28). Intriguingly, there were two putative ELK1 binding sites within the promoter of *PLXDC2* (Supplementary Fig. S7). Moreover, TCGA analysis showed a strong correlation between the expression of *ELK1* and *PLXDC2* genes in HPV(−) HNSCC cohorts (Fig. 7F), but not HPV(+) tumors (Supplementary Fig. S8). We then depleted ELK1 in HNSCC cells using lentiviral-mediated shRNAs. ELK1 ablation markedly inhibited *PLXDC2* expression in CAL27 cells (Fig. 7G). To investigate whether the specific binding of ELK1 to the *PLXDC2* promoter was required for the transcriptional activation of *PLXDC2*, we performed qPCR-ChIP assays. This analysis revealed the specific occupancy of ELK1 at the *PLXDC2* promoter-binding sites in HNSCC cells (Fig. 7H). Increased ELK1 binding amount was detected on the *PLXDC2* promoter when c-Met was overexpressed in CAL27 cells (Fig. 7H) and the binding of ELK1 in these cells was significantly weaker in comparison with cells treated with SCH772984 (Fig. 7I). Furthermore, more ELK1 proteins were found on the *PLXDC2* promoter in radioresistant CAL27 cells relative to parental cells, while blocking c-Met signaling in these cells by SU11274 dramatically abrogated the binding of ELK1 to the *PLXDC2* promoter (Fig. 7J). These results were confirmed in radioresistant and parental HN6 cells (Supplementary Fig. S9), indicating that ELK1 facilitates *PLXDC2* transcriptional expression in the context of activation of c-Met-ERK1/2 signaling in HNSCC cells.

## Discussion

HNSCC resistance to radiotherapy remains a critical barrier to improving the survival of patients diagnosed with locally advanced, unresectable cancers or resected disease requiring postoperative radiation. To mitigate or even overcome radioresistance, it is imperative to understand and subsequently target the mechanisms of radioresistance that are associated with cell death evasion and tumor aggression. There is the cross-talk between RTKs and cancer radioresistance; thus, we parallelly determined the phosphorylation alterations of RTKs in radioresistant and parental CAL27 cells using the Proteome Profiler Human Phospho-RTK Array Kit. Elevated p-c-Met phosphorylation levels, along with decreased phosphorylation of nine RTKs (FGFR1, FGFR3, EphA1, EphA2, EphB2, ROR1, MCSFR, DDR1, and Flt-3), were identified in radioresistant CAL27 cells. Decreased RTK phosphor-activation is unexpected to be observed in radioresistant cancer cells, which may vary depending on the cell context. Most importantly, we identified a novel c-Met–mediated molecular basis of radioresistance, which has the broad applicability in HNSCC cells.

One study has demonstrated that PLXDC2 is highly expressed in stromal cells and its cross-talk with tumor-associated macrophages contributes to cancer biology by inducing the EMT process (29). One follow-up investigation further showed that *PLXDC2* is a critical gene associated with the TME, and its upregulation correlates with advanced clinical stages and predicts a shorter OS of patients with gastric cancer (30). Combining RNA-seq with qRT-PCR analysis, we initially found and confirmed that *PLXDC2* is a novel downstream target of c-Met. We then illustrated that PLXDC2 plays a critical role in HNSCC cell plasticity through promoting EMT and the emergence of dedifferentiated cells with CSC-like properties. This protein was upregulated in radioresistant HNSCC cells compared with their nonresistant counterparts. Elevated PLXDC2 in response to activation of c-Met signaling further confers radioresistance in HNSCC cells during radiotherapy. PLXDC2 upregulation was also observed in primary postradiotherapy tumor tissues collected from patients with radioresistant HNSCC, suggesting that our research findings have a strong clinical relevance. Mechanistically, the present study demonstrates that c-Met upregulates PLXDC2 via ERK1/2 signaling. Elevated PLXDC2 expression in radioresistant HNSCC cells is mediated by c-Met-ERK1/2 activation, which phosphorylates the downstream effector ELK1, promoting it to translocate into the nucleus and bind to the *PLXDC2* gene promoter to initiate the gene transcription. To our knowledge, this is the first study elucidating the molecular link between c-Met and PLXDC2 in cancer cells.

c-Met is among the most well-studied RTKs out of 14 RTK families (31). It is not surprising that c-Met is the molecular determinant that confers radioresistance of HNSCC given its profound roles in cell survival, proliferation, and migration/invasion (32). In this study, we unraveled that increased c-Met phosphor-activation represents an essential mechanism by which HNSCC cells acquire radioresistance. Hyperactivation of c-Met is the primary event toward HNSCC cell plasticity and stemness. Our results were consistent with previous observations by Lim and colleagues and Sun and colleagues who suggested that c-Met activation contributed to producing stem-like HNSCC population (33, 34). Inactivating c-Met by genetic knockdown or pharmacologic treatment not only reverses the EMT process but also diminishes the CD44^+^CD133^−^ CSC population in radioresistant HNSCC cells, leading to an overall reduction in tumor development and progression. Our current study, using both *in vitro* and *in vivo* models, provides solid preclinical support for the potential of c-Met targeting strategies that can be used to overcome radioresistance in HNSCC. This notion was also suggested recently by a research group in Germany (23).

A considerable body of evidence supports the hypothesis that radiotherapy preferentially eliminates nontumorigenic cells and enriches CSCs by “awakening” them to initiate proliferation and differentiation (4). CSCs constitute only a small percentage (0.05%–1%) of tumor cells within a tumor mass. Still, they are the “seeds” of cancer associated with an aggressive tumor phenotype characterized by increased cell survival, migration, invasion, metastatic capacity, treatment resistance, and tumor recurrence (10). As CSCs have innately higher radioresistance than their differentiated cancer cell counterparts, they develop a series of aggressive phenotypes, which is considered one of the leading causes of cancer relapse and metastasis after radiotherapy (10). Recently, several studies have demonstrated that radiotherapy has the side effect of generating new CSCs from normal and neoplastic non-stem cells, leading to an increase in the number of CSCs (4, 10, 35, 36). It appears that more and “awakened” CSCs are in tumors when they develop radioresistance, which plays a crucial role in tumor relapse and metastasis after radiotherapy. Our data support this notion and further demonstrate that the cells exhibiting EMT exist in the CSC population and contribute to radioresistance in HNSCC.

c-Met hyperactivation has been observed in numerous neoplasms, including HNSCC. Prolonged or continuous activity of this RTK results in cancer cell survival and aggressiveness related to the development and progression of cancer. c-Met inhibitors can be classified into three groups: small-molecule tyrosine kinase inhibitors (SU11274, crizotinib, tivantinib, cabozantinib, and foretinib), mAbs against c-Met (onartuzumab), and against the ligand hepatocyte growth factor (ficlatuzumab and rilotumumab; ref. 37). SU11274 was identified as a small molecule, ATP competitive inhibitor of the catalytic activity of c-Met. It has been reported that SU11274 significantly suppressed the phosphorylation of c-Met and constrained the growth of colorectal carcinoma cell-derived xenograft tumors in nude mice (38). We compared the inhibitory effects of three c-Met inhibitors, SU11274, foretinib, and crizotinib, on c-Met activation in CAL27 cells at the same dose and found that SU11274 is the most effective in impairing c-Met phosphorylation. We also demonstrated that SU11274 has the great potential to enhance cellular sensitivity to radiation in 3D cell cultures and preclinical tumor-bearing animals. Thus far, the use of c-Met inhibitors in HNSCC has been restricted to the recurrent and metastatic disease setting in the absence of radiation (39). Despite the evidence that c-Met inhibition reduces cell proliferation, tumor growth and angiogenesis (40–42), the inhibition of c-Met clinically has not resulted in significant observed benefit for patients with advanced HNSCC thus far. Knowledge gained from our study suggests that the combination of SU11274 and radiotherapy represents a promising regimen to improve the efficacy of conventional radiotherapy by inhibiting c-Met–mediated cancer cell aggressiveness. This observation is supported by a study which showed combinations of c-Met–targeted therapy and radiotherapy could enhance treatment efficacy in breast cancer (43).

In summary, this study delineates a previously unrecognized mechanism underpinning c-Met-PLXDC2–mediated radioresistance, opening the door for considering possible combinatorial approaches of c-Met inhibitors with radiotherapy in HNSCC.

## Supplementary Material

Supplementary Table ST1 The primer sequences for qRT-PCR assays.

Supplementary Figure S1 Effect of IR on viability of radioresistant and parental CAL27 and HN6 cells determined by alamarBlue assays on Day 5 after IR.

Supplementary Figure S2 Cell proliferative potential between radioresistant and parental CAL27 and HN6 cells determine by MTT assays three days after incubation.

Supplementary Figure S3 Inhibitory effect of three c-Met inhibitors on c-Met phosphorylation in CAL27 cells. CAL27 cells were treated with 2.5 µM SU11274, foretinib or crizotinib for 24 hours, followed by Western blotting analysis.

Supplementary Figure S4 Validation the expression levels of top five most upregulated genes (identified from RNA-seq) in radioresistant CAL27 (A) and HN6 (B) cells using qRT-PCR. *p<0.05; **p<0.01.

Supplementary Figure S5 c-Met-PLXDC2 signaling regulates EMT-associated invasion in HN6 cells.

Supplementary Figure S6 Effect of PLXDC2 knockdown on colony formation of radioresistant CAL27 and HN6 cells determined on Day 12 following 4 Gy IR exposure

Supplementary Figure S7 The putative ELK1 binding sites (the sequences are in red with indicated location) on the PLXDC2 promoter region upstream of the start codon for the full-length protein.

Supplementary Figure S8 The correlation between the expression of ELK1 and PLXDC2 genes illustrated in scatter plot from the TCGA HPV(+) HNSCC cohort (n=98).

Supplementary Figure S9 Elevated PLXDC2 expression in radioresistant HN6 cells is mediated by c-Met-activated ERK1/2-ELK signaling.
