# Supplementary material for: Adaptive c-Met-PLXDC2 Signaling Axis Mediates Cancer Stem Cell Plasticity to Confer Radioresistance-associated Aggressiveness in Head and Neck Cancer
Source: Cancer Res Commun. 2023 Apr 19;3(4):659–71. doi: 10.1158/2767-9764.CRC-22-0289 (PMC10114932; doi:10.1158/2767-9764.CRC-22-0289)
Supplement: Supplementary Table ST1 — The primer sequences for qRT-PCR assays. [file crc-22-0289-s01.docx]

**Supplementary Table S1. The primer sequences for qRT-PCR assays.**

| **Gene Name** | **Sequence (5' to 3')** |
| --- | --- |
| DIO2-F | TCGATGCCTACAAACAGGTGAA |
| DIO2-R | AGGGCTGGCAAAGTCAAGAA |
| PLXDC2 -F | GGTAGACACGAACCGAGCAA |
| PLXDC2-R | TGTGGTCTGTATCCTCCTCGAT |
| NCKAP5-F | CAGCGGTGGCAGTAATAGTGA |
| NCKAP5-R | GTTGTCTCAATTTCTGGGGCA |
| BMP7-F | TTCGTCAACCTCGTGGAACA |
| BMP7-R | ACGTCTCATTGTCGAAGCGT |
| XCR1-F | CAGAGAAGCACCATTTCTGCC |
| XCR1-R | CCAGATGGCAGGGACGTTT |
