# Supplementary material for: Adaptive c-Met-PLXDC2 Signaling Axis Mediates Cancer Stem Cell Plasticity to Confer Radioresistance-associated Aggressiveness in Head and Neck Cancer
Source: Cancer Res Commun. 2023 Apr 19;3(4):659–71. doi: 10.1158/2767-9764.CRC-22-0289 (PMC10114932; doi:10.1158/2767-9764.CRC-22-0289)
Supplement: Supplementary Figure S1 — Effect of IR on viability of radioresistant and parental CAL27 and HN6 cells determined by alamarBlue assays on Day 5 after IR. [file crc-22-0289-s02.docx]

**
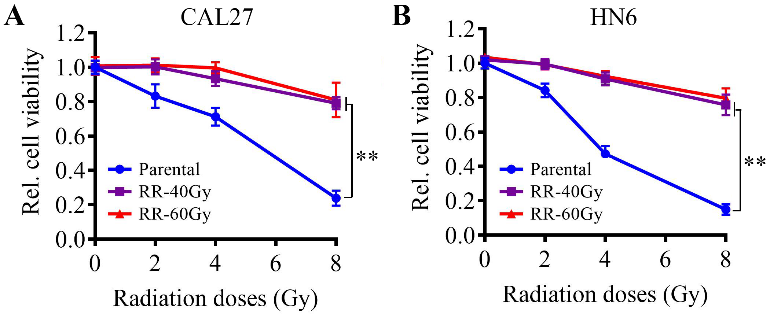
**

**Supplementary Figure S1.** Effect of IR on viability of radioresistant and parental CAL27 and HN6 cells determined by alamarBlue assays on Day 5 after IR. ***p*<0.01.
