# Supplementary material for: Adaptive c-Met-PLXDC2 Signaling Axis Mediates Cancer Stem Cell Plasticity to Confer Radioresistance-associated Aggressiveness in Head and Neck Cancer
Source: Cancer Res Commun. 2023 Apr 19;3(4):659–71. doi: 10.1158/2767-9764.CRC-22-0289 (PMC10114932; doi:10.1158/2767-9764.CRC-22-0289)
Supplement: Supplementary Figure S3 — Inhibitory effect of three c-Met inhibitors on c-Met phosphorylation in CAL27 cells. CAL27 cells were treated with 2.5 µM SU11274, foretinib or crizotinib for 24 hours, followed by Western blotting analysis. [file crc-22-0289-s04.docx]

**
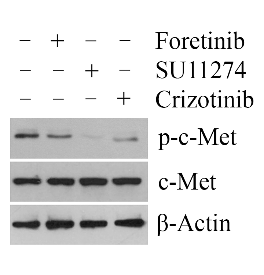
**

**Supplementary Figure S3.**  Inhibitory effect of three c-Met inhibitors on c-Met phosphorylation in CAL27 cells. CAL27 cells were treated with 2.5 µM SU11274, foretinib or crizotinib for 24 hours, followed by Western blotting analysis.
