# Supplementary material for: Adaptive c-Met-PLXDC2 Signaling Axis Mediates Cancer Stem Cell Plasticity to Confer Radioresistance-associated Aggressiveness in Head and Neck Cancer
Source: Cancer Res Commun. 2023 Apr 19;3(4):659–71. doi: 10.1158/2767-9764.CRC-22-0289 (PMC10114932; doi:10.1158/2767-9764.CRC-22-0289)
Supplement: Supplementary Figure S4 — Validation the expression levels of top five most upregulated genes (identified from RNA-seq) in radioresistant CAL27 (A) and HN6 (B) cells using qRT-PCR. *p<0.05; **p<0.01. [file crc-22-0289-s05.docx]

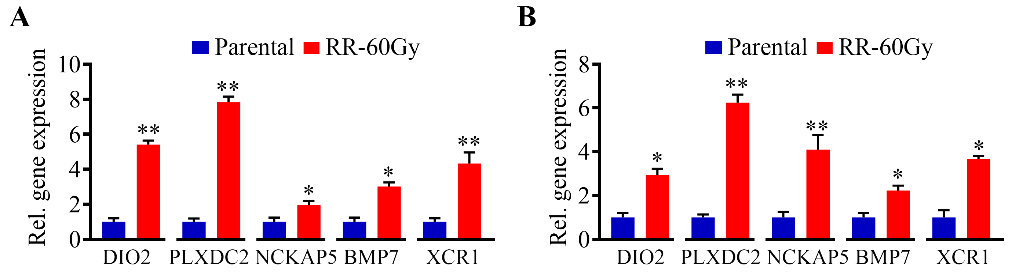


**Supplementary Figure S4. Validation the expression levels of top five most upregulated genes (identified from RNA-seq) in radioresistant CAL27 (A) and HN6 (B) cells using qRT-PCR.** **p*<0.05; ***p*<0.01.
