# Supplementary material for: Adaptive c-Met-PLXDC2 Signaling Axis Mediates Cancer Stem Cell Plasticity to Confer Radioresistance-associated Aggressiveness in Head and Neck Cancer
Source: Cancer Res Commun. 2023 Apr 19;3(4):659–71. doi: 10.1158/2767-9764.CRC-22-0289 (PMC10114932; doi:10.1158/2767-9764.CRC-22-0289)
Supplement: Supplementary Figure S5 — c-Met-PLXDC2 signaling regulates EMT-associated invasion in HN6 cells. [file crc-22-0289-s06.docx]

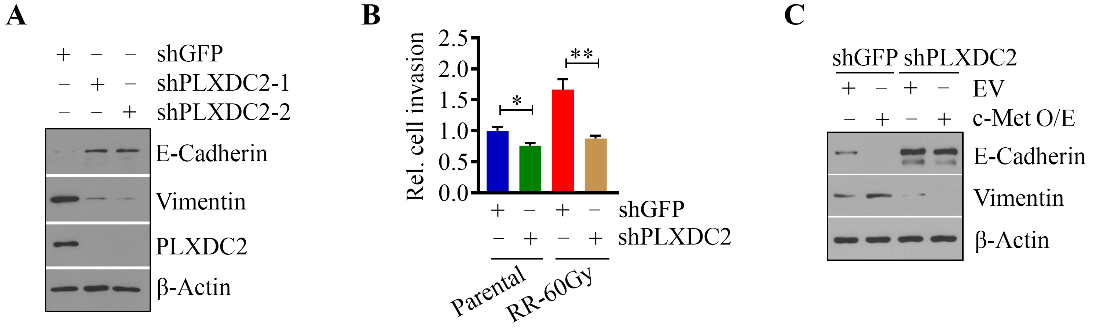


**Supplementary Figure S5. c-Met-PLXDC2 signaling regulates EMT-associated invasion in HN6 cells.** (A) Protein levels of E-cadherin and vimentin in PLXDC2 knockdown and control HN6 cells. (B) Effect of PLXDC2 knockdown on cell invasion in radioresistant and parental HN6 cells. (C) Protein levels of E-cadherin and vimentin in c-Met overexpression and control HN6 cells with or without PLXDC2 knockdown.
