# Supplementary material for: Adaptive c-Met-PLXDC2 Signaling Axis Mediates Cancer Stem Cell Plasticity to Confer Radioresistance-associated Aggressiveness in Head and Neck Cancer
Source: Cancer Res Commun. 2023 Apr 19;3(4):659–71. doi: 10.1158/2767-9764.CRC-22-0289 (PMC10114932; doi:10.1158/2767-9764.CRC-22-0289)
Supplement: Supplementary Figure S6 — Effect of PLXDC2 knockdown on colony formation of radioresistant CAL27 and HN6 cells determined on Day 12 following 4 Gy IR exposure [file crc-22-0289-s07.docx]

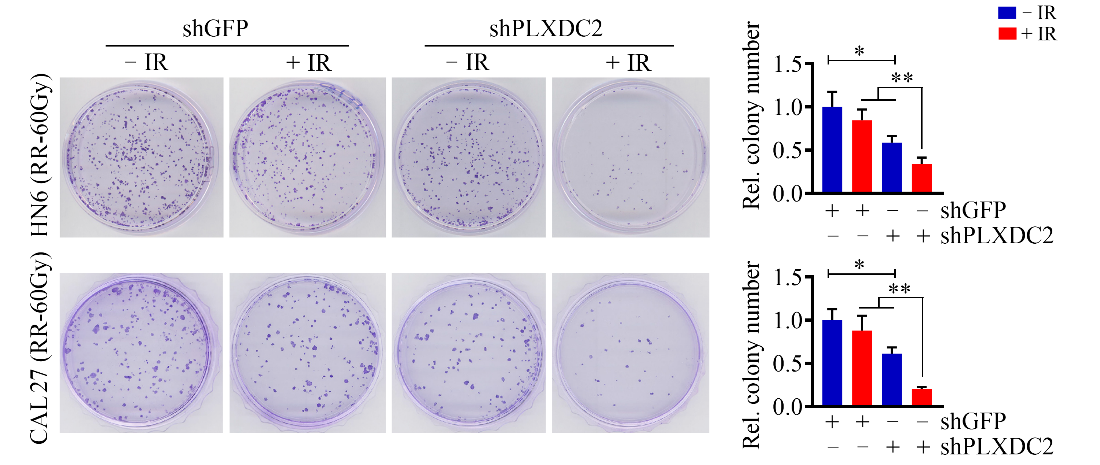


**Supplementary Figure S6. Effect of PLXDC2 knockdown on colony formation of radioresistant CAL27 and HN6 cells determined on Day 12 following 4 Gy IR exposure.** Representative images and quantitative data from three independent experiments are shown in the left and right panels, respectively. **p*<0.05; ***p*<0.01.
