# Supplementary material for: Adaptive c-Met-PLXDC2 Signaling Axis Mediates Cancer Stem Cell Plasticity to Confer Radioresistance-associated Aggressiveness in Head and Neck Cancer
Source: Cancer Res Commun. 2023 Apr 19;3(4):659–71. doi: 10.1158/2767-9764.CRC-22-0289 (PMC10114932; doi:10.1158/2767-9764.CRC-22-0289)
Supplement: Supplementary Figure S7 — The putative ELK1 binding sites (the sequences are in red with indicated location) on the PLXDC2 promoter region upstream of the start codon for the full-length protein. [file crc-22-0289-s08.docx]

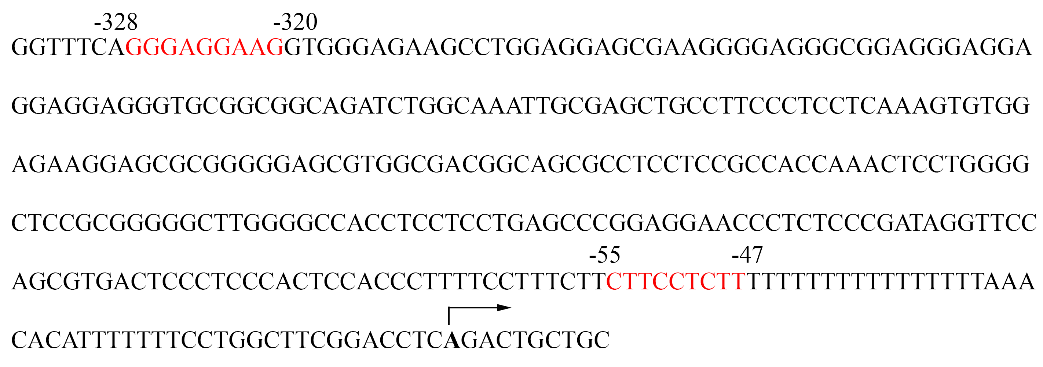


**Supplementary Figure S7.** The putative ELK1 binding sites (the sequences are in red with indicated location) on the PLXDC2 promoter region upstream of the start codon for the full-length protein.
