# Supplementary material for: Adaptive c-Met-PLXDC2 Signaling Axis Mediates Cancer Stem Cell Plasticity to Confer Radioresistance-associated Aggressiveness in Head and Neck Cancer
Source: Cancer Res Commun. 2023 Apr 19;3(4):659–71. doi: 10.1158/2767-9764.CRC-22-0289 (PMC10114932; doi:10.1158/2767-9764.CRC-22-0289)
Supplement: Supplementary Figure S8 — The correlation between the expression of ELK1 and PLXDC2 genes illustrated in scatter plot from the TCGA HPV(+) HNSCC cohort (n=98). [file crc-22-0289-s09.docx]

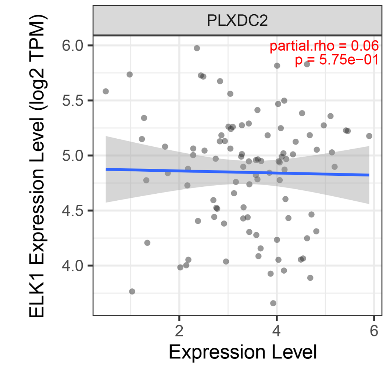


**Supplementary Figure S8.** The correlation between the expression of ELK1 and PLXDC2 genes illustrated in scatter plot from the TCGA HPV(+) HNSCC cohort (n=98).
