# Supplementary material for: Adaptive c-Met-PLXDC2 Signaling Axis Mediates Cancer Stem Cell Plasticity to Confer Radioresistance-associated Aggressiveness in Head and Neck Cancer
Source: Cancer Res Commun. 2023 Apr 19;3(4):659–71. doi: 10.1158/2767-9764.CRC-22-0289 (PMC10114932; doi:10.1158/2767-9764.CRC-22-0289)
Supplement: Supplementary Figure S9 — Elevated PLXDC2 expression in radioresistant HN6 cells is mediated by c-Met-activated ERK1/2-ELK signaling. [file crc-22-0289-s10.docx]

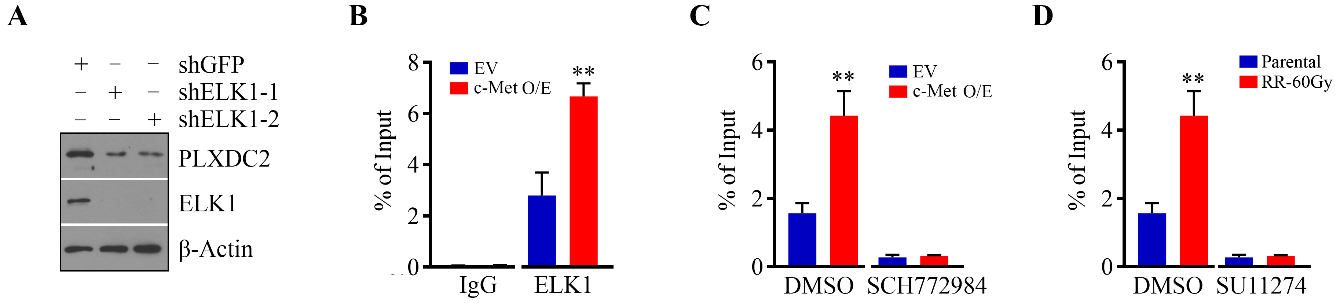


**Supplementary Figure S9. Elevated PLXDC2 expression in radioresistant HN6 cells is mediated by c-Met-activated ERK1/2-ELK signaling.** (A) Effect of ELK1 knockdown on PLXDC2 expression in HN6 cells. (B) The binding of ELK1 protein on the PLXDC2 gene promoter in c-Met overexpression and control HN6 cells determined by ChIP-qPCR assays. (C) Effect of SCH772984 on ELK1 binding on the PLXDC2 gene promoter in c-Met overexpression and control HN6 cells determined by ChIP-qPCR assays. (D) Effect of SU11274 on ELK1 binding on the PLXDC2 gene promoter in radioresistant and parental HN6 cells determined by ChIP-qPCR assays. In (B), (C) and (D), quantitative data are obtained from three independent experiments. ***p*<0.01.
